# Supplementary material for: Apparent stability masks underlying change in a mule deer herd with unmanaged chronic wasting disease
Source: Commun Biol. 2022 Jan 11;5:15. doi: 10.1038/s42003-021-02951-z (PMC8752592; doi:10.1038/s42003-021-02951-z)
Supplement: Supplementary file 2 — Reporting Summary [file 42003_2021_2951_MOESM2_ESM.pdf]

nature portfolio

Corresponding author(s): Michael W. Miller

Last updated by author(s): Nov 21, 2021

Reporting Summary

Nature Portfolio wishes to improve the reproducibility of the work that we publish. This form provides structure for consistency and transparency in reporting. For further information on Nature Portfolio policies, see our Editorial Policies and the Editorial Policy Checklist.

Statistics

For all statistical analyses, confirm that the following items are present in the figure legend, table legend, main text, or Methods section.

n/a	Confirmed	
☐	☒	The exact sample size (*n*) for each experimental group/condition, given as a discrete number and unit of measurement
☐	☒	A statement on whether measurements were taken from distinct samples or whether the same sample was measured repeatedly
☐	☒	The statistical test(s) used AND whether they are one- or two-sided<br>*Only common tests should be described solely by name; describe more complex techniques in the Methods section.*
☐	☒	A description of all covariates tested
☐	☒	A description of any assumptions or corrections, such as tests of normality and adjustment for multiple comparisons
☐	☒	A full description of the statistical parameters including central tendency (e.g. means) or other basic estimates (e.g. regression coefficient) AND variation (e.g. standard deviation) or associated estimates of uncertainty (e.g. confidence intervals)
☐	☒	For null hypothesis testing, the test statistic (e.g. *F*, *t*, *r*) with confidence intervals, effect sizes, degrees of freedom and *P* value noted<br>*Give P values as exact values whenever suitable.*
☒	☐	For Bayesian analysis, information on the choice of priors and Markov chain Monte Carlo settings
☒	☐	For hierarchical and complex designs, identification of the appropriate level for tests and full reporting of outcomes
☒	☐	Estimates of effect sizes (e.g. Cohen's *d*, Pearson's *r*), indicating how they were calculated

Our web collection on statistics for biologists contains articles on many of the points above.

Software and code

Policy information about availability of computer code

Data collection	No software used to collect data.
Data analysis	Microsoft Excel; WinPepi (Abramson, J.H. WINPEPI updated: computer programs for epidemiologists, and their teaching potential. Epidemiologic Perspectives & Innovations 2011, 8:1); Program NOREMARK (White, G. C. NOREMARK: population estimation from mark-resighting surveys. Wildl. Soc. Bull. 24, 50-52, 1996). [Analysis note: coauthor JPR is a biometrician for Colorado Parks & Wildlife.]

For manuscripts utilizing custom algorithms or software that are central to the research but not yet described in published literature, software must be made available to editors and reviewers. We strongly encourage code deposition in a community repository (e.g. GitHub). See the Nature Portfolio guidelines for submitting code & software for further information.

Data

Policy information about availability of data

All manuscripts must include a data availability statement. This statement should provide the following information, where applicable:

- Accession codes, unique identifiers, or web links for publicly available datasets
- A description of any restrictions on data availability
- For clinical datasets or third party data, please ensure that the statement adheres to our policy

Data files available from the Dryad Digital Data Repository: https://doi.org/10.5061/dryad.fbg79cnw6.

Field-specific reporting

Please select the one below that is the best fit for your research. If you are not sure, read the appropriate sections before making your selection.

☐ Life sciences ☐ Behavioural & social sciences ☒ Ecological, evolutionary & environmental sciences

For a reference copy of the document with all sections, see nature.com/documents/nr-reporting-summary-flat.pdf

Ecological, evolutionary & environmental sciences study design

All studies must disclose on these points even when the disclosure is negative.

Study description

Our main study objectives were to estimate chronic wasting disease (CWD) prevalence and population demographic parameters (i.e., abundance, age and sex composition) in the free-ranging Table Mesa mule deer herd residing in southwestern Boulder, Colorado USA, with the intention of comparing contemporary field data to those from a prior study (ca. 2005-08) as a basis for documenting trends and evaluating long(ish)-term disease impacts in this deer herd.

A written study plan detailing and justifying approaches was reviewed and approved by our institutional animal care & use committee prior to commencing field work. All animals were sampled and individually marked; a subset was marked with GPS/VHF telemetry collars to facilitate study of habitat use, survival, and movement analysis for a companion project. Telemetered animals were monitored remotely & causes of mortality investigated in the field.

We measured contemporary CWD prevalence and compared to data from 2005 (Miller et al. 2008) to assess the 10+ year trend in a highly infected herd. Population size and composition also were estimated via mark-resight and compared with results from previous estimates in this area made using the same counting methods and routes. Initial (unexpected) findings led us to explore & report additional comparisons (e.g., genetic & survival) intended to help identify potential processes driving the broader patterns observed.

Target sample sizes for each objective were as shown below. See "Sampling strategy" section for additional details.

Table. Primary research objectives and target sample sizes for each.

Objective	Sample size ♂	Sample size ♀	Justification
- Prevalence	56	56	sufficient to detect doubling of prevalence among females & 2-fold difference between sexes
- Abundance (MR)	10	20	sufficient to yield a CV of ~3% in a population of 300 animals
- Habitat selection	10	40	sufficient to distribute collars throughout the Table Mesa herd range

Research sample

We studied free-ranging mule deer (Odocoileus hemionus) in the "Table Mesa" herd residing in southwestern Boulder, Colorado USA. We captured 100 deer -- 54 females, 46 males -- during November 2018-February 2019, avoiding capture and sampling of juveniles. [Note: We suspended captures prior to reaching the targets for prevalence comparisons because preliminary data showed a priori hypotheses on expected differences would not be supported even with additional data.]

We distributed captures throughout the ~23 km^2 study area described by Miller et al. (2008; details farther below) to minimize spatial disparities in comparing contemporary and past data, and to assure marks were widely distributed for December ground counts to estimate deer abundance. Sample distribution ultimately was dictated by the distribution of deer within the study area on days when captures were attempted.

Mule deer are a natural host of CWD, which has individual- and herd-level effects in natural systems. We chose this study site based on existence of historical data for comparison as a basis for testing hypotheses describing prevalence and demographic trends, evaluating long-term disease impacts, and informing future management decisions in an unhunted herd known to be heavily infected with prion disease.

Sampling strategy

We captured deer randomly and opportunistically, avoiding only juveniles and those individuals already sampled. Based on abundance estimates, our sample included nearly half of the non-juvenile deer in the Table Mesa herd & thus seemed sufficiently representative.

Target sample sizes related to epidemiology questions were established based on testing a priori hypotheses about expected prevalence patterns. Assuming a one-sided test, a sample size of 56 (vs. 61 from prior sampling in 2005-06) would have been sufficient to detect a doubling in prevalence among females (from ~23% in 2005-06 to ~46% in 2018-19). We chose a one-sided test because a marked decline in prevalence among females seemed unlikely based on expected epidemic behavior. Similarly, 56 males would have allowed us to detect a two-fold difference between sexes with reasonable confidence. (Tables provided in the original study plan available upon request.) The target sample also would have readily detected a doubling in prevalence among males, but that outcome seemed unlikely based on maximum prevalence observed elsewhere. We noted at the time of planning this study that little or no evidence of a change in prevalence over the last ~13 years also would be an important finding in the context of assessing potential changes in abundance over the same time period because the reported rates already were remarkably high.

For abundance estimates, we used data from 2005-2007 to calculate a minimum sample size. Annual point estimates for deer abundance (2005-2008) ranged from 238 to 286. Using Bowden's estimator, marking 25 deer in a population of 300 would yield an expected CV of about 3% for a combined abundance estimate provided at least 7 resighting occasions were completed. The

estimated numbers of females, males, and fawns were generated from Program MARK based on count data.

Data collection	Field methods generally followed those from the earlier Table Mesa study (Miller et al. 2008).<br><br>Briefly, we pursued deer on foot and darted them opportunistically, delivering sedative combinations intramuscularly via projectile syringe. Premixed immobilization drug combinations included either nalbuphine (N; 0.9 mg/kg) or butorphanol (B; 0.5 mg/kg) combined with azaperone (A; 0.2 mg/kg) and medetomidine (M; 0.2 mg/kg), with standard total doses for respective combinations based on an estimated mass of 70 kg (average drug volume per animal was 1.3 ml NMA, 1.4 ml BAM). We collected rectal mucosa biopsies to determine CWD infection status as described by Wolfe et al. (2007). We also collected whole blood and marked all deer with individually identifiable ear tags and some with telemetry (n=51) or visual identification (n=12) collars. Ages were estimated visually to the nearest year via tooth replacement and wear patterns. To antagonize sedation upon completion of handling and sampling, each deer received 5 mg atipamezole/mg M administered, injected intramuscularly.<br><br>Formalin-fixed tissue biopsies were processed and analyzed by immunohistochemistry (IHC) at the Colorado State University Veterinary Diagnostic Laboratory (Fort Collins, Colorado USA; CSUVDL) for evidence of CWD-associated prion (PrP^CWD) accumulations using monoclonal antibody F99/97.6.1 (VMRD Inc., Pullman, Washington, USA) and methods described previously. Biopsies were evaluated microscopically and classified as positive (infected) or not detected (negative) based on PrP^CWD presence or absence. We included only biopsies with at least 5 lymphoid follicles in our analyses of prevalence data, thereby excluding 14 females and five males.<br><br>We used DNA extracted from buffy coat aliquots (n=99) to screen for the presence of serine (S) and/or phenylalanine (F) at PRNP gene codon 225, classifying individuals as 225SS, 225SF, or 225FF using methods described by Jewell et al. (2005).
Timing and spatial scale	Captures were done during daylight hours from November 2018-February 2019. The capture schedule was flexible to accommodate weather and personnel. (A list of specific capture dates can be generated upon request.) Seven ground counts were done during daylight hours in December 2018 (toward the end of the breeding season) to assure adult male deer could be readily counted. (Male deer tend to retreat to smaller, more secluded groups after breeding and this can lead to underestimating their abundance if counts are done too late in winter.)<br><br>We distributed captures throughout the ~23 km^2 Table Mesa study area described by Miller et al. (2008) to minimize spatial disparities in comparing contemporary and past data, and to assure marks were widely distributed for December ground counts to estimate deer abundance. Sample distribution ultimately was dictated by the distribution of deer within the study area on the days when capture crews were in the field.
Data exclusions	We included only results from biopsies with at least 3 lymphoid follicles in our analyses of prevalence data to ensure adequate probability of detecting prion infection if present. This a priori criterion excluded biopsy data from seven females and two males from some analyses.<br><br>One animal was excluded from genetic comparisons because no blood was collected at the time of capture.
Reproducibility	We attempted to the extent feasible to replicate the methods used in our earlier study of this herd, but repeating the current study was not feasible. Laboratory tests were run with appropriate positive and negative controls; key laboratory personnel were the same individuals involved in the earlier Table Mesa study, thereby offering added consistency. Age estimates were periodically checked by experienced independent observers. Ground counts were repeated to assure sufficient precision in resulting estimates, and personnel were rotated among routes to avoid potential observer bias.
Randomization	Deer were captured randomly and opportunistically. We planned to capture an equal number of male and female deer but males were less abundant & thus fewer were available. Sampling and marking were done in the field absent knowledge on disease status. Grouping for subsequent comparisons & hypothesis tests (e.g., by PRNP genotype) were based on laboratory results.
Blinding	Laboratory analyses (e.g., interpreting biopsies, genotyping) were done by laboratory professionals blinded to the provenance of individual samples.

Did the study involve field work? ☒ Yes ☐ No

Field work, collection and transport

Field conditions	Conditions varied, typical of winter in Colorado's northern Front Range. These were noted on individual capture cards but were not central to our study and thus were not summarized. The National Center for Atmospheric Research is located in the study area & would be a source of detailed weather records should these be needed.
Location	Table Mesa, Boulder, Colorado USA: The ~23 km^2 ''Table Mesa'' study area included low-elevation (1,660–2,050 m) mule deer range at an urban-open space interface including private and public lands (City of Boulder Open Space and Mountain Parks) bounded by Baseline Road on the north, Colorado Highway 93 on the east, South Boulder Creek on the south, and the Flatiron Mountain front to the west. Our study area encompassed native habitats and urban landscapes developed within those habitats, best characterized as mountain shrub habitat interspersed with mixed forb and grassland openings and timbered patches dominated by ponderosa pine

	(Pinus ponderosa). Mule deer lived throughout the study area, sometimes close to human dwellings.
Access & import/export	The collaborating City of Boulder owned much of the deer-occupied land within the study area & granted permission to capture & monitor deer on open space property. Private and other restricted-access properties were entered only after securing permission from owners or their representatives. City field crews sometimes operated independently under a Scientific Collecting License issued by Colorado Parks & Wildlife.<br><br>No import/export occurred.
Disturbance	The Table Mesa area is heavily used by humans, and consequently mule deer in this area are relatively habituated to human activity. Nonetheless, approaching groups of deer and capturing individuals did cause temporary disturbance. We minimized handling times and moved on when groups became unapproachable. We also established criteria for suspending capture if ambient temperatures became or were forecast to be unacceptably high or low.<br><br>Drugging, handling, sampling, and marking was undoubtedly disturbing to affected individuals. We handled captured animals as quickly as possible and used antagonists to facilitate rapid recovery from immobilization. Some components in the drug combinations used also provided somewhat extended calming and pain relief post capture.

Reporting for specific materials, systems and methods

We require information from authors about some types of materials, experimental systems and methods used in many studies. Here, indicate whether each material, system or method listed is relevant to your study. If you are not sure if a list item applies to your research, read the appropriate section before selecting a response.

Materials & experimental systems

n/a	Involved in the study
☐	☒ Antibodies
☒	☐ Eukaryotic cell lines
☒	☐ Palaeontology and archaeology
☐	☒ Animals and other organisms
☒	☐ Human research participants
☒	☐ Clinical data
☒	☐ Dual use research of concern

Methods

n/a	Involved in the study
☒	☐ ChIP-seq
☒	☐ Flow cytometry
☒	☐ MRI-based neuroimaging

Antibodies

Antibodies used	monoclonal antibody F99/97.6.1 used in immunohistochemistry
Validation	Approved for prion diagnostics in the US; also see: Spraker, T. R., et al. Validation of monoclonal antibody F99/97.6.1 for immunohistochemical staining of brain and tonsil in mule deer (Odocoileus hemionus) with chronic wasting disease. J. Vet. Diagn. Invest. 14, 3-7 (2002).

Animals and other organisms

Policy information about studies involving animals; ARRIVE guidelines recommended for reporting animal research

Laboratory animals	none
Wild animals	We captured and sampled free-ranging, non-juvenile mule deer (Odocoileus hemionus). This included 54 females and 46 males. Mean (± 95% CI) estimated ages among captured deer were 4.4 (± 0.5) yr for females and 3.5 (± 0.3) yr for males.<br><br>Briefly, we pursued deer on foot and darted them opportunistically, delivering sedative combinations intramuscularly via projectile syringe. Premixed immobilization drug combinations included either nalbuphine (N; 0.9 mg/kg) or butorphanol (B; 0.5 mg/kg) combined with azaperone (A; 0.2 mg/kg) and medetomidine (M; 0.2 mg/kg), with standard total doses for respective combinations based on an estimated mass of 70 kg (average drug volume per animal was 1.3 ml NMA, 1.4 ml BAM).<br><br>All captured animals were released on site after sampling and marking. To antagonize sedation upon completion of handling and sampling, each deer received 5 mg atipamezole/mg M administered, injected intramuscularly.
Field-collected samples	In this context, we interpreted the term "sample" as a reference to live study subjects collected in the field and subsequently held under captive conditions for experimentation. We collected biological specimens and monitored study subjects in situ.<br><br>[Note: We did collect biological specimens -- whole blood, formalin-fixed tissue biopsies -- in the field. These were analyzed as described in a previous section on "Data collection".]

Ethics oversight	Field procedures were reviewed and approved by the Colorado Parks & Wildlife Animal Care and Use Committee (file 14-2018).

Note that full information on the approval of the study protocol must also be provided in the manuscript.
